# Oligomerization Strategy of D-A-Type Conjugated Molecules for Improved NIR-II Fluorescence Imaging

**DOI:** 10.3390/polym15163451

**Published:** 2023-08-18

**Authors:** Tongqing Zou, Yu Liu, Xinyue Zhang, Lu Chen, Qinqin Xu, Yancheng Ding, Ping Li, Chen Xie, Chao Yin, Quli Fan

**Affiliations:** State Key Laboratory of Organic Electronics and Information Displays, Institute of Advanced Materials (IAM), Nanjing University of Posts and Telecommunications, 9 Wenyuan Road, Nanjing 210023, China; b20050315@njupt.edu.cn (T.Z.); 1221066933@njupt.edu.cn (Y.L.); 1222067233@njupt.edu.cn (X.Z.); 1221066930@njupt.edu.cn (L.C.); iamqinqinxu@163.com (Q.X.); 1222067232@njupt.edu.cn (Y.D.); iampingli@njupt.edu.cn (P.L.)

**Keywords:** near-infrared-II window, fluorescence imaging, conjugated polymer, oligomerization strategy

## Abstract

Fluorescence imaging (FI) in the second near-infrared (NIR-II) window has emerged as a promising imaging method for cancer diagnosis because of its superior properties such as deep penetration depth and high signal-to-background ratio. Despite the superiorities of organic conjugated nanomaterials for NIR-II FI, the issues of low fluorescence quantum yield, weak metabolic capability, undefined molecular structure for conjugated polymers, weak light-harvesting ability, short emission wavelength, and high synthetic complexity for conjugated small molecules still remain to be concerned. We herein propose an oligomerization strategy by facilely adjusting the oligomerization time to balance the advantages and disadvantages between conjugated polymers and small molecules, obtaining the candidate (CO1, oligomerization time: 1 min) with the optimal NIR-II optical performance. Then the CO1 is further prepared into water-dispersed nanoparticles (CON1) via a nanoprecipitation approach. By virtue of the suitable size, excellent NIR-II optical properties, low toxicity, and strong cell-labeling ability, the CON1 is successfully employed for in vivo NIR-II imaging, permitting the real-time visualization of blood vascular system and tumors with high sensitivity and resolution. This work thus not only provides a personalized organic conjugated nano-agent for NIR-II FI, but also highlights the molecular strategy for the development of organic conjugated systems with optimal performance for bio-imaging.

## 1. Introduction

Fluorescence imaging (FI) conducted over near-infrared-II (NIR-II) optical windows has emerged as a promising imaging technique for cancer diagnosis because of its superior properties such as deep penetration depth, high spatial resolution, and high sensitivity [1,2,3]. In order to amplify the NIR-II fluorescence signal in living subjects to facilitate the clinical translation of NIR-II FI, various optical agents, such as inorganic nanomaterials [4], organic small molecular dyes [5], and conjugated polymers/oligomers [6,7,8], have been developed to act as contrast agents for NIR-II FI. In general, inorganic noble metal nanoparticles (e.g., Ag_2_S [9], Ag_2_Te [10], and AgAuSe [11]) have a relatively high fluorescence quantum yield; however, such materials inevitably suffer from the issues of long-term biotoxicity because of the heavy metal ions and difficulties in rapid clearance. Although organic small molecular dyes show good biocompatibility, the inferior photostability and tumor accumulation capability restrict their further in vivo applications [12]. Therefore, the development of new contrast agents with reliable biosafety, high photostability, and strong tumor accumulation ability is highly desired for efficient NIR-II FI in living bodies.

Organic conjugated materials including conjugated polymers (CPs) and conjugated small molecules (CSMs) represent a class of promising optical probes for bio-imaging owing to their outstanding advantages, such as strong light-harvesting ability, high photostability, and low toxicity [13,14,15,16,17]. Furthermore, the structural diversity allows the development of CPs or CSMs with tunable light response range, even in the NIR-II optical region [18,19]. Thus, such materials have been widely explored and utilized for NIR-II FI [20,21,22], NIR-II photoacoustic imaging [23,24], NIR-II photothermal therapy [25,26,27], and NIR-II photodynamic therapy [28]. For example, by linking thiadiazoloquinoxaline (TQX) and diketopyrrolopyrrole (DPP) units via Stille polymerization, Pu et al. developed a CP with D-A1-D-A2 architecture [23]. Because of the strong electron-withdrawing ability of TQX, the developed CP exhibited strong absorption in the NIR-II region, permitting effective NIR-II photoacoustic imaging in vivo. After transferring the CP to aqueous media via the nanoprecipitation method, the obtained nanoparticles showed uniform spherical morphology with an average diameter of 54 nm. Interestingly, the nanoparticles exhibited identical photoacoustic intensity at 750 (NIR-I) and 1064 nm (NIR-II), making it rational to compare the photoacoustic performance between these two wavelengths. Comprehensive investigations using chicken breast tissue verified the superiority of NIR-II photoacoustic imaging in practical use mainly due to the reduced photon scattering of NIR-II light in living tissues. In addition, Liu et al. reported a thienoisoindigo derivative-based D-A type CP for NIR-II photothermal therapy [27]. The strong intramolecular charge transfer resulting from the D-A feature endowed the CP with remarkable NIR absorption even to the NIR-II region. Upon water soluble modification and the HA decoration, biocompatible and tumor-targeted nanoparticles were fabricated, which exhibited high photothermal conversion efficiency under 1064 nm light irradiation. In vitro and in vivo studies demonstrated that the prepared nanoparticles could not only kill cancer cells but also ablate the tumor in the presence of the NIR-II light irradiation, providing an effective application demonstration of CPs for NIR-II photothermal therapy against cancer. It is worth noting that CPs and CSMs have their own advantages and disadvantages in practical applications. For CPs, the most significant superiority is the strong light-harvesting capacity and high photostability. Furthermore, it is very convenient to construct the π-conjugated backbone of CPs via typical coupling polymerizations (e.g., Stille coupling [29], Suzuki coupling [30], and Sonogashira coupling [31]), and the target polymers can be easily obtained by convenient purification approaches, such as precipitation and Soxhlet extraction. However, owing to the large molecular weight, CPs are prone to accumulate in the liver and spleen, which need a long time to be cleared out from the body. Furthermore, CPs generally have relatively low NIR-II fluorescence quantum yields, which is assigned to the accelerated nonradiative decay by the vibrational overlap between the ground state and excited state of the NIR-II CPs [32]. In addition, the undefined molecular structure of CPs makes it difficult to achieve good repeatability. For CSMs, they are more likely to have the ability of biodegradation and easy clearance owing to the small molecular weight, demonstrating the more optimal biosafety. However, the relatively short emission wavelength resulting from the insufficient degree of conjugation limits the imaging applications of CSMs in the NIR-II window. Furthermore, column chromatography is generally adopted to purify the CSMs, which is complicated and time-consuming. Therefore, the development of new organic conjugated materials and relevant synthetic strategies is urgently needed to advance the development of NIR-II FI in the field of biomedicine.

**Figure 1:**
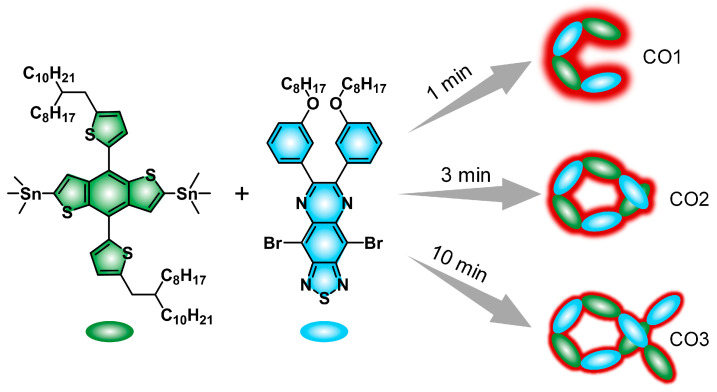
The synthetic illustration of CO1, CO2, and CO3. A growing halo from CO3 to CO1 represented the gradually amplified NIR-II fluorescent brightness.

Herein, an oligomerization strategy by facilely adjusting the reaction time is proposed to balance the advantages and disadvantages between CPs and CSMs, aiming to optimize the candidate with the best NIR-II optical performance for in vivo imaging. Benzodithiophene (BDT) and TQX are chosen as the electron donor (D) and acceptor (A), respectively, to construct the organic conjugated backbone through the Stille coupling reaction (Figure 1). By adjusting the reaction time (1 min, 3 min, and 10 min), conjugated oligomers (COs) with different molecular weights are synthesized first, followed by a comparative study of their NIR-II optical performances. The optimal CO1 is fabricated into water-dispersed nanoparticles (CON1) that are successfully employed for angiography and tumor imaging with high sensitivity and resolution. Excellent NIR-II optical performance and reliable biosafety offer a promising potential of CON1 for future theranostic applications.

## 2. Experimental Section

### 2.1. Materials and Characterizations

(4,8-bis(5-(2-octyldodecyl)thiophen-2-yl)benzo [1,2-b:4,5-b′]dithiophene-2,6-diyl)bis(trimethylstannane) and 4,9-dibromo-6,7-bis(3-(octyloxy)phenyl)-[1,2,5]thiadiazolo[3,4-g]quinoxaline were obtained from SunaTech Inc. (Suzhou, China). All the other chemicals, such as tris(dibenzylideneacetone)dipalladium, tris(2-methylphenyl)-phosphine, toluene, methanol, poly(ethylene glycol)-*block*-poly(propylene glycol)-*block*-poly(ethylene glycol), and tetrahydrofuran (THF) were purchased from Sigma-Aldrich (St. Louis, MI, USA) and used directly.

The Bruker Ultra Shield Plus NMR instrument (Bruker Corporation, Saarbrucken, Germany, 400 MHz) was employed to collect the ^1^H NMR data. Gel permeation chromatography (GPC) analysis was performed on Shim-pack GPC-80X columns with THF as the eluent and polystyrenes as the standard. Shimadzu UV-3600 UV-vis-NIR spectrophotometer was used to collect the absorption spectra. NIR-II fluorescence spectra were recorded on a commercial NIR-II spectrophotometer (Fluorolog 3, Horiba, Tokyo, Japan) equipped with an 808 nm diode laser, a 1064 nm diode laser, and an InGaAs NIR detector. TEM images were obtained in a JEOL JEM-2100 transmission electron microscope (JEOL Ltd., Tokyo, Japan). A small drop of the nanoparticle dispersion was placed on a copper grid coated with a carbon film and then the grid was used for TEM measurements after drying at room temperature. DLS measurement was conducted in the 90 Plus particle size analyzer (Brookhaven Instruments, Holtsville, NY, USA). MTT assay was carried out using a PowerWave XS/XS2 microplate spectrophotometer (BioTek, Winooski, VT, USA). Cell imaging was carried out on a confocal laser scanning microscope (CLSM) and magnified with Fluoview software (Olympus, FV1000, Tokyo, Japan). In vivo NIR-II fluorescence imaging was conducted on an NIR-II imaging system (Wuhan Grand-imaging Technology Co., Ltd., Wuhan, China).

### 2.2. Synthesis of COs

All the COs were synthesized through a Stille coupling reaction with various reaction time periods (1 min, 3 min, 10 min). Taking CO1 as an example, (4,8-bis(5-(2-octyldodecyl)thiophen-2-yl)benzo[1,2-b:4,5-b′]dithiophene-2,6-diyl)bis(trimethylstannane) (20.0 mg), 4,9-dibromo-6,7-bis(3-(octyloxy)phenyl)-[1,2,5]thiadiazolo[3,4-g]quinoxaline (20.0 mg), tris(dibenzylideneacetone)dipalladium (8.0 mg), and tris(2-methylphenyl)-phosphine (4.0 mg) were placed in a 10 mL flask, then 4.0 mL of toluene was added into the mixture under N_2_ protection. Subsequently, the mixture was subjected to the freeze–pump–thaw process three times. Then the mixture was vigorously stirred at 100 °C for 1 min. At last, the mixture was concentrated and dropped into methanol to precipitate the crude product, which was washed with methanol three times and dried. The CO2 and CO3 were synthesized via a similar process of CO1, adjusting the reaction time to 3 and 10 min, respectively. The structures of all the COs were confirmed by ^1^H NMR and GPC measurements.

### 2.3. Fabrication of CONs

All the CONs were fabricated via the nanoprecipitation method. For the CON1, 1.0 mg CO1 and 20.0 mg poly(ethylene glycol)-*block*-poly(propylene glycol)-*block*-poly(ethylene glycol) were dissolved in THF (1.0 mL), which was rapidly injected into pure water (9.0 mL) under ultrasonication. Then the THF was removed by N_2_ blowing on the solution surface under stirring at room temperature, yielding the water-dispersed nanoparticles, CON1. By changing the precursor to CO2 and CO3, the CON2 and CON3 were prepared with the same procedure. The prepared CONs were stored in a 4 °C refrigerator for further use.

### 2.4. Fabrication of CON1-FITC

Next, 10 mg poly(ethylene glycol)-*block*-poly(propylene glycol)-*block*-poly(ethylene glycol) was dissolved in 1 mL THF, to which the THF solution of FITC (5 μL, 1 mg/mL) and CO1 (100 μL, 1 mg/mL) was added. Then the mixture was rapidly injected into 5 mL of pure water under ultrasonication. Finally, the residual THF was removed by N_2_ blowing, yielding the water-dispersed nanoparticles, CON1-FITC.

### 2.5. MTT Assay

The biocompatibility of CON1 was assessed by testing the cell viability via MTT assay. 3T3 fibroblasts were seeded in 96-well plates (2 × 10^4^ cells/mL). After incubating in DMEM medium at 37 °C in a 5% CO_2_ humidified atmosphere for 24 h, CON1 was added into the medium with various concentrations (0, 1, 2, 4, 8, 16, and 32 μg/mL) to treat the cells for another 24 h. Then the medium was removed and the cells were washed with PBS, and the MTT solution (100 μL, 0.5 mg/mL) was added to each well. After incubation for 3 h at 37 °C, the MTT medium solution was removed, and the formazan crystals were dissolved by dimethylsulfoxide (DMSO) (200 μL). Then the absorbance value was recorded at 490 nm using a microplate reader to assess the cell viability. The absorbance of the untreated cells was used as a control with its absorbance as the reference value for calculating 100% cellular viability.

### 2.6. Cellular Uptake

4T1 cancer cells were cultured in the incubator at 37 °C for 24 h. Then the CON1-FITC was added to the cells with a concentration of 32 μg/mL. After the cells were incubated for 4 h or 6 h, the culture medium was removed and the cells were washed with PBS. The cells without CON1-FITC treatment were used as the control. Next, 150 μL DAPI staining solution was added to each confocal dish, and the dish was kept in the dark for 10 min. After that, the cells were washed with PBS twice. 4T1 cells were imaged by a confocal laser scanning microscopy (CLSM) with 488 nm laser excitation and 405 nm laser excitation for FITC and DAPI visualization, respectively.

### 2.7. Animal Model

BALB/c nude mice (15–20 g body weight) were used for blood vessel imaging. BALB/c mice (20–22 g body weight) were employed for tumor imaging. To establish the tumor model, each mouse was subcutaneously injected with 200 μL of 4T1 cell suspension (5 × 10^6^ cells). All the mice experiments were performed in accordance with the guidelines of the Laboratory Animal Center of Jiangsu KeyGEN Biotech Corp., Ltd. (Nanjing, China) and approved by the Animal Ethics Committee of Simcere BioTech Corp., Ltd. (Nanjing, China). The mice were anesthetized using isoflurane if necessary.

### 2.8. NIR-II Fluorescence Imaging In Vivo

For blood vessel imaging, BALB/c nude mice were anesthetized using 2% isoflurane in oxygen, and the CON1 (1.0 mg/mL, 125 μL) was intravenously injected through the tail vein using a microsyringe. The NIR-II fluorescence imaging was performed on the NIR-II imaging system. Fluorescence images of the mice were acquired at the time point of 3 min after CON1 administration. For tumor imaging, the tumor-bearing mice were intravenously injected with CON1 (150 μL, 0.2 mg/mL) and then imaged at different time points of post-injection (0, 2, 6, 12, 24, 36, 48, and 60 h) on the NIR-II fluorescence imaging system. For ex vivo fluorescence imaging, mice were sacrificed at 60 h post-injection, and the heart, liver, spleen, lung, kidney, and tumor were harvested for ex vivo fluorescence imaging to analyze the bio-distribution of the CON1.

### 2.9. Data Analysis

NIR-II fluorescence intensities were measured by region-of-interest (ROI) analysis using the NIR-II living imaging system. Results were expressed as the mean ± SD unless otherwise stated. All statistical calculations were conducted using OriginPro 8.5 software.

## 3. Results and Discussion

All the COs (CO1, CO2, CO3) were synthesized via a Pd-catalyzed Stille coupling reaction (Figure 1). BDT and TQX were chosen as the building blocks to construct the organic conjugated backbone because such a D-A alternately linked structure generally has a red-shifted absorption/emission band, which is beneficial for bio-imaging conducted over longer wavelength even to the NIR-II region [33]. The structures of all the COs were characterized via ^1^H NMR (Figures S1–S3, Supporting Information) and gel permeation chromatography (GPC) (Figure S4 and Table S1, Supporting Information) measurements. The resonance peaks located at 6.5–8.0 ppm are attributed to the hydrogen signals from the benzene ring and thiophene. The resonance peak that appeared at ~3.7 ppm is assigned to the hydrogen signal of -O-CH_2_- from the TQX segment. This verified the successful linkage between BDT and TQX. Furthermore, the bulky resonance peaks ranging from 0.8 to 1.8 ppm are hydrogen signals from the alkyl chain. GPC curves indicated the gradually decreased elution time from CO1 to CO3, and the GPC results demonstrated the gradually increased molecular weight from CO1 to CO3. These data revealed the increased oligomerization degree by prolonging the reaction time from 1 min to 10 min. All these data clearly confirmed the successful synthesis of COs.

**Figure 2:**
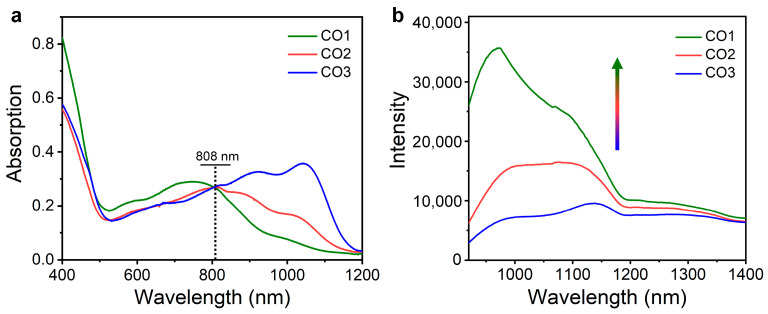
Optical properties of the COs. (**a**) The absorption spectra of COs, in which the absorbance values at 808 nm were adjusted to the same level; (**b**) the fluorescence spectra of COs upon 808 nm laser excitation.

All the COs can be dissolved well in commonly used organic solvents such as tetrahydrofuran (THF). The optical properties of COs were studied through measuring the absorption and emission spectra of the THF solution. The CO1 showed an absorption peak at 750 nm with a weak shoulder peak at ~1000 nm in the NIR region (Figure 2a). In contrast, CO2 and CO3 exhibited significant red shift of the absorption band, whose absorption peaks appeared at 810 and 1040 nm, respectively (Figure 2a). Such results should be attributed to the fact that the increased oligomerization degree effectively narrowed the energy gap of COs, thus red shifting the absorption band. When the absorbance at 808 nm (Ab808) were fixed to the same level, the fluorescence spectra of the COs were recorded under 808 nm light excitation. All the COs showed intense emission over 1000 nm (Figure 2b), indicating the promising potential for in vivo NIR-II FI. Impressively, CO1 exhibited the highest NIR-II fluorescence intensity, followed by CO2 and CO3, and the intensity of the highest emission peak of CO1 was 2.2- and 3.7-fold enhancement compared with that of CO2 and CO3, respectively (Figure 2b). The weakest NIR-II fluorescence of CO3 should be attributed to two aspects. First, based on the energy gap law, the narrower the CPs’ energy gap, the greater the nonradiative decay rate, thus leading to the reduced fluorescence. Second, the strong self-absorption effect over 1000 nm further weakened the NIR-II emission of CO3. Therefore, the NIR-II optical performance of organic conjugated systems could be optimized through modulating the oligomerization degree of COs.

**Figure 3:**
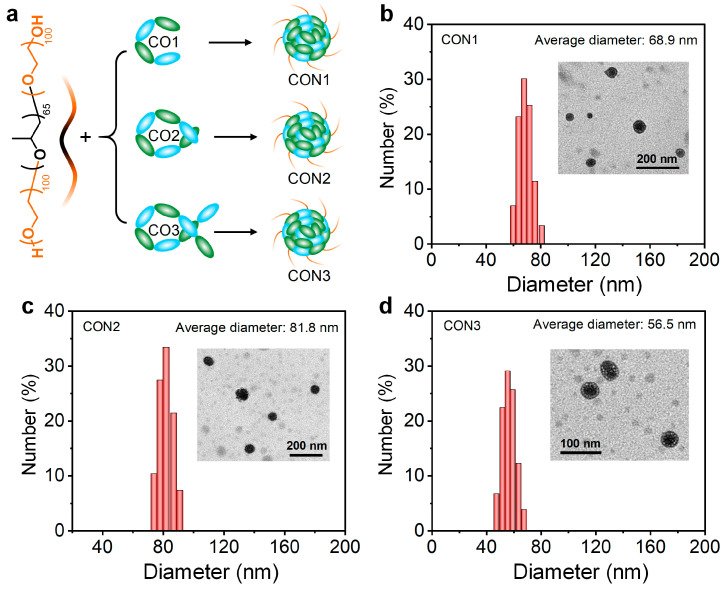
(**a**) The fabrication process of CONs via nanoprecipitation method; TEM (inset) and DLS results of the CON1 (**b**), CON2 (**c**), and CON3 (**d**).

Subsequently, all the COs were prepared into water-dispersed nanoparticles (CON1, CON2, and CON3) assisted by the amphiphilic block copolymer poly(ethylene glycol)-*block*-poly(propylene glycol)-*block*-poly(ethylene glycol) via nanoprecipitation approach (Figure 3a). Transmission electron microscopy (TEM) showed the spherical morphology for all the CONs (Figure 3b–d). Dynamic light scattering (DLS) indicated the average diameter of 68.9, 81.8, and 56.5 nm for CON1, CON2, and CON3, respectively (Figure 3b–d). Some slight differences in the nanoparticles size were observed between TEM and DLS measurements, which should be attributed to the shrinkage of CONs during the drying process. Because CO1 had the optimal NIR-II performance among all the COs, the CON1 fabricated from CO1 was employed for further bioresearch.

**Figure 4:**
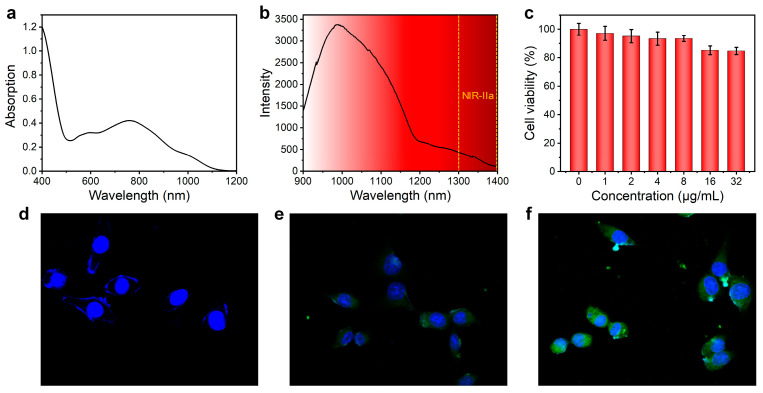
(**a**) The absorption spectrum of CON1 in aqueous media; (**b**) the fluorescence spectrum of CON1 aqueous dispersion under 808 nm laser excitation; (**c**) cell viability tests of NIH 3T3 fibroblasts treated with CON1 under various concentrations. Confocal imaging of 4T1 cancer cells received no treatments (**d**), CON1-FITC treatment for 4 h (**e**) or 6 h (**f**). The blue and green fluorescence are attributed to the DAPI and CON1-FITC signals, respectively.

The CON1 showed similar absorption profile compared with its precursor, CO1 (Figure 4a). To closely compare their optical properties, we normalized the absorption spectra of CO1 and CON1 with respect to the peak maximum around 750 nm, and the results are shown in Figure S5. From the data, we can see that there is a slight absorption improvement around 1000 nm for CON1 relative to CO1. Furthermore, the spectrum of CON1 showed a slight red-shift compared with that of CO1. These results indicated the aggregation state of CO1 inside the nanoparticles. Upon 808 nm laser excitation, the fluorescence spectrum of CON1 showed an emission peak at 986 nm and tailed to 1300–1400 nm (NIR-IIa region) (Figure 4b), which was favorable for achieving high fidelity imaging with an improved signal-to-noise ratio (SNR). Such excellent optical performance provided high potential of CON1 for in vivo imaging.

The biosafety and cellular uptake were then assessed. Although the cell viability of NIH 3T3 fibroblasts showed a slight decrease with the increased concentration of CON1 by MTT assay, almost all the NIH 3T3 fibroblasts were alive (survival rate: ~85%) after the treatment of CON1 with a relatively high concentration (32 μg/mL) (Figure 4c). Such results indicated the negligible influence of CON1 on the metabolism of normal cells. In order to visually observe the cellular uptake process of CON1, the fluorescein isothiocyanate (FITC) was co-encapsulated into the nanoparticles when fabricating CON1, yielding the CON1-FITC. It clearly presented that the cytoplasm of 4T1 cells emitted remarkable green fluorescence after CON1-FITC treatment for 4 h (Figure 4e), revealing the effective internalization of CON1-FITC by cancer cells. With the incubation time extended to 6 h, much stronger fluorescence from 4T1 cells was observed (Figure 4f), indicating that such cellular uptake behavior was time dependent. In contrast, no green fluorescence signals were detected and only the cell nuclei were identified from the cells without any treatments (Figure 4d). All these results manifested the prominent cellular uptake ability of CON1, which laid the foundation for tumor theranostics in living subjects.

**Figure 5:**
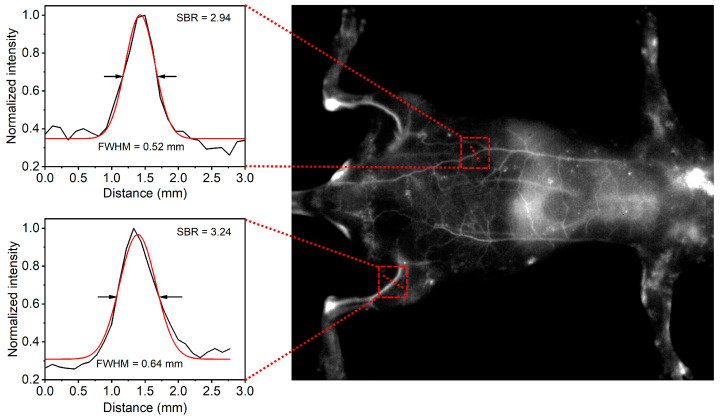
The blood vessels imaging of mice after intravenous injection of CON1 for 3 min. The NIR-II fluorescence signals were collected using a 1200 nm long pass filter under 808 nm laser excitation. Left: Cross-sectional intensity profile along the red dash line across one of the main blood vessels or hind limb vessels in the right image.

**Figure 6:**
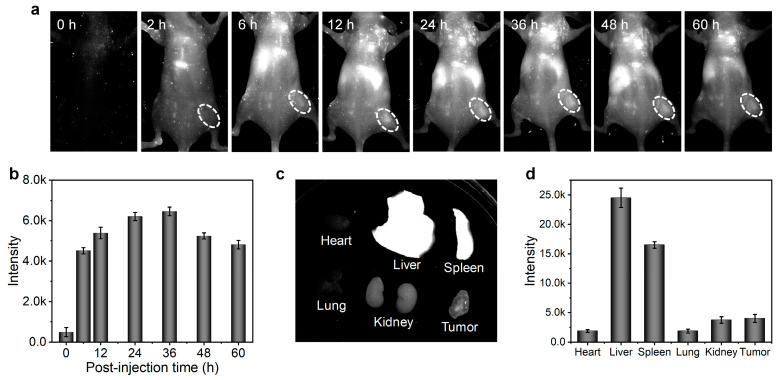
(**a**) In vivo tumor imaging of mice at different time points after intravenous injection of CON1. The NIR-II fluorescence signals were collected using a 980 nm long pass filter under 808 nm laser excitation. The white dash circle indicated the tumor region; (**b**) the quantification of tumor signals at different time points after injection. The NIR-II FI (**c**) and signal quantification (**d**) of the main organs and tumor excised from mice at 60 h post-injection.

Next, the CON1-assisted NIR-II FI in living animals was performed. After intravenous injection of CON1 for 3 min, the whole vascular network of the mouse was lit up with high contrast (Figure 5). Specifically, the main and branched vessels from the abdomen region were clearly delineated with high spatial resolution, and the imaging SNR of one of the main vessels reached 2.94 (Figure 5). Furthermore, the hind limb vessels were identified sharply with the SNR of 3.24 (Figure 5). At this time point, the liver was slightly lit up by NIR-II fluorescence (Figure 5), inferring a small accumulation of CON1 in the liver at this stage. Furthermore, the in vivo tumor imaging was conducted. Before systemic administration, nearly no NIR-II fluorescence signals were detected from the mice (Figure 6a), indicating the low background interference of NIR-II imaging. Upon intravenous injection of CON1, the tumor site was lit up and the signal intensity gradually increased over time (Figure 6a,b). At the time point of 36 h post-injection, the tumor signal reached maximum, which exhibited 12.9-fold enhancement relative to that of background (Figure 6b). After that, tumor signals gradually weakened. The mice were sacrificed at 60 h post-injection, and the major organs were excised to analyze the biodistribution of CON1. Liver had the strongest NIR-II signal, followed by spleen (Figure 6c,d). This demonstrated the hepatobiliary metabolism process of CON1 in living mice. The tumor showed stronger NIR-II signals than the heart, lung, and kidney (Figure 6d), verifying the ideal accumulation ability of CON1 in the tumor site. These results confirmed the outstanding imaging performance of CON1 in the living body, thereby making it a promising candidate for diagnostic applications.

## 4. Conclusions

In conclusion, an oligomerization strategy by adjusting the reaction time was proposed to balance the advantages and disadvantages between CPs and CSMs. By using BDT and TQX as the electron donor and electron acceptor, respectively, three oligomers (CO1, CO2, and CO3) were synthesized via Pd-catalyzed Stille coupling reaction with various reaction time periods (1 min, 3 min, and 10 min), which were characterized by ^1^H NMR and GPC measurements. The photophysical studies indicated that the CO1 produced from the oligomerization process for 1 min had the optimal NIR-II fluorescent performance among the COs when their absorption values at 808 nm were adjusted to the same level. Thus, the CO1 was further fabricated into water-dispersed nanoparticles (CON1) for biological research. Owing to the superiorities of suitable size, strong NIR-II emission, and reliable biocompatibility, the CON1 was successfully utilized for in vivo NIR-II FI. It clearly demonstrated that the whole vascular network of the mouse was lit up and delineated with high spatial resolution, and specifically, the imaging SNR of one of the main vessels and the hind limb vessels reached 2.94 and 3.24, respectively. Furthermore, the tumor profile of living mice could be depicted with high sensitivity, revealing the potential for cancer diagnosis. This work not only synthesized a series of NIR-II emissive COs and optimized the best-performing candidate for in vivo imaging, but also provided a molecular strategy to explore high-efficiency organic conjugated materials for phototheranostics.

## Data Availability

The data presented in this study are available on request from the corresponding author.

## Supplementary Materials

The following supporting information can be downloaded at: https://www.mdpi.com/article/10.3390/polym15163451/s1, Figure S1: ^1^H NMR spectrum of the CO1; Figure S2: ^1^H NMR spectrum of the CO2; Figure S3: ^1^H NMR spectrum of the CO3; Figure S4: GPC curves of the CO1, CO2, and CO3; Figure S5: Normalized absorption spectra of CO1 and CON1; Table S1: GPC results of the CO1, CO2, and CO3.
