# Supplementary material for: Oligomerization Strategy of D-A-Type Conjugated Molecules for Improved NIR-II Fluorescence Imaging
Source: Polymers (Basel). 2023 Aug 18;15(16):3451. doi: 10.3390/polym15163451 (PMC10460014; doi:10.3390/polym15163451)
Supplement: Supplementary file 1 [file polymers-15-03451-s001.zip › polymers-2539158-supplementary.pdf]

Supplementary Materials

Oligomerization strategy of D-A-type conjugated molecules for improved NIR-II fluorescence imaging

Tongqing Zou §, Yu Liu §, Xinyue Zhang, Lu Chen, Qinqin Xu, Yancheng Ding, Ping Li, Chen Xie *, Chao Yin * and Quli Fan *

State Key Laboratory of Organic Electronics and Information Displays & Institute of Advanced Materials (IAM), Nanjing University of Posts and Telecommunications, 9 Wenyuan Road, Nanjing 210023, China

* Correspondence: Correspondence: iamcxie@njupt.edu.cn (Chen Xie); iamcyin@njupt.edu.cn (Chao Yin); iamqlfan@njupt.edu.cn (Quli Fan) .

§ These authors contributed equally (Tongqing Zou and Yu Liu)

1. Supporting figures

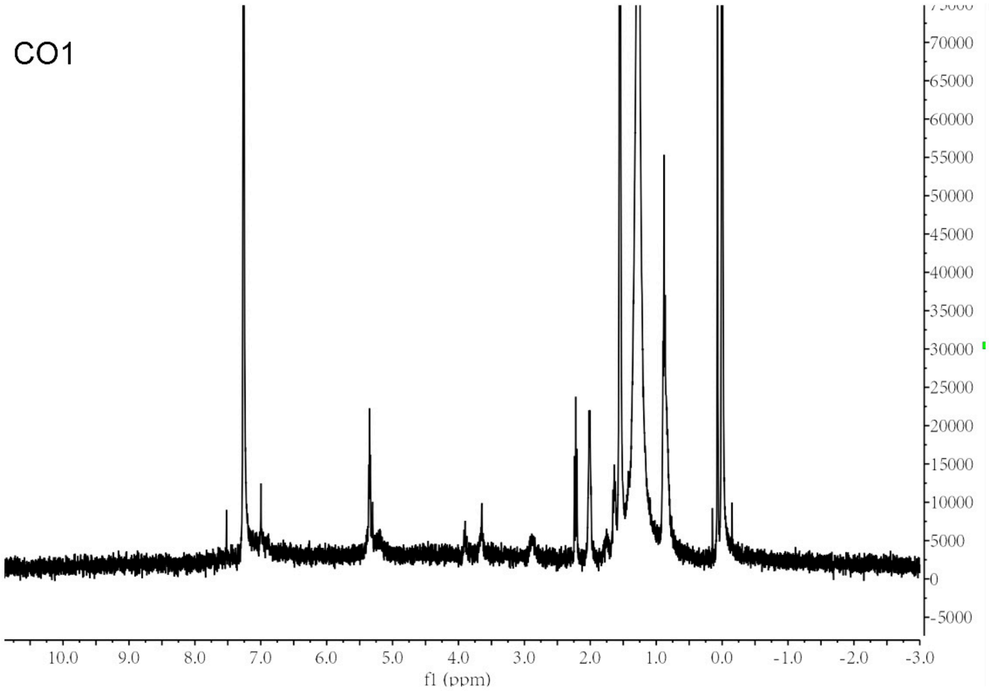

Figure S1. ^{1}H NMR spectrum of the CO1. $CDCl_3$ was used as the solvent.

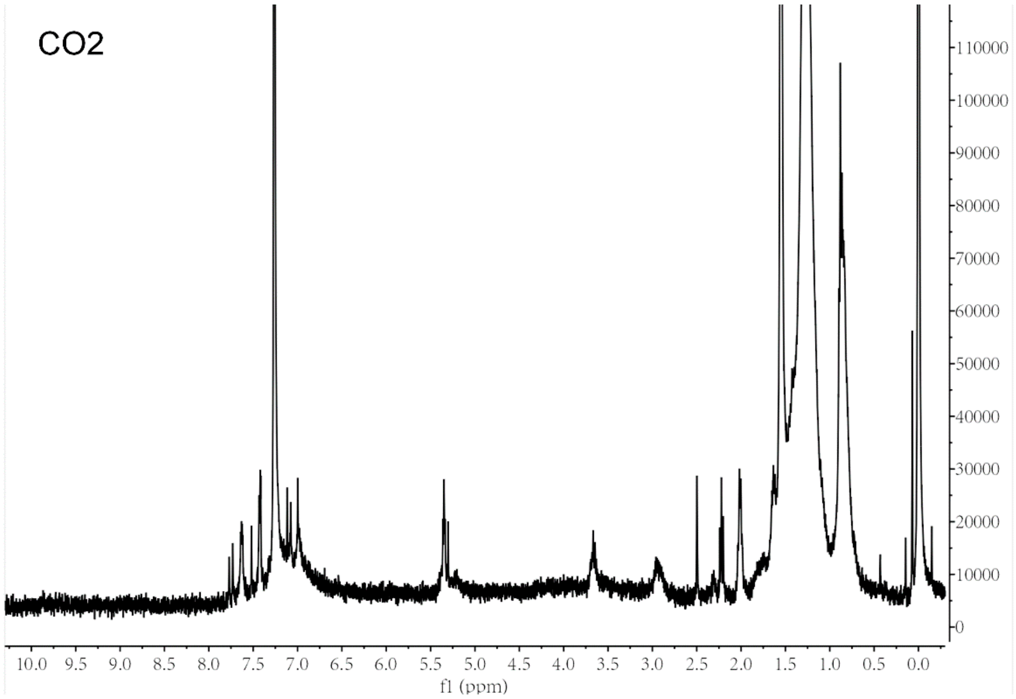

Figure S2. ^{1}H NMR spectrum of the CO2. $CDCl_3$ was used as the solvent.

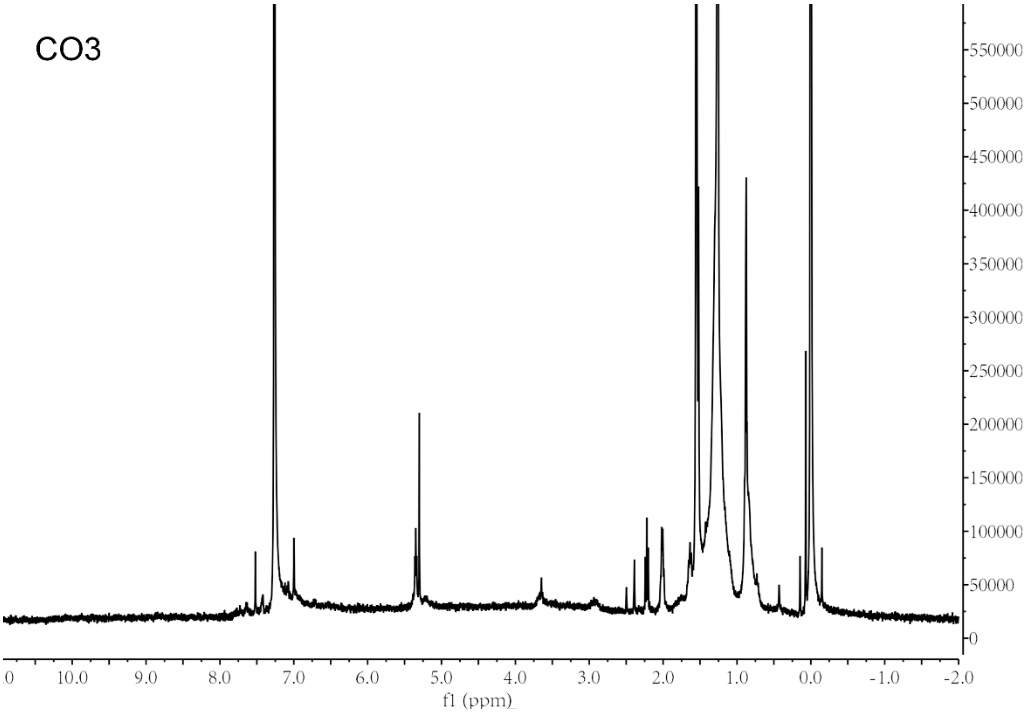

Figure S3. ^{1}H NMR spectrum of the CO3. $CDCl_3$ was used as the solvent.

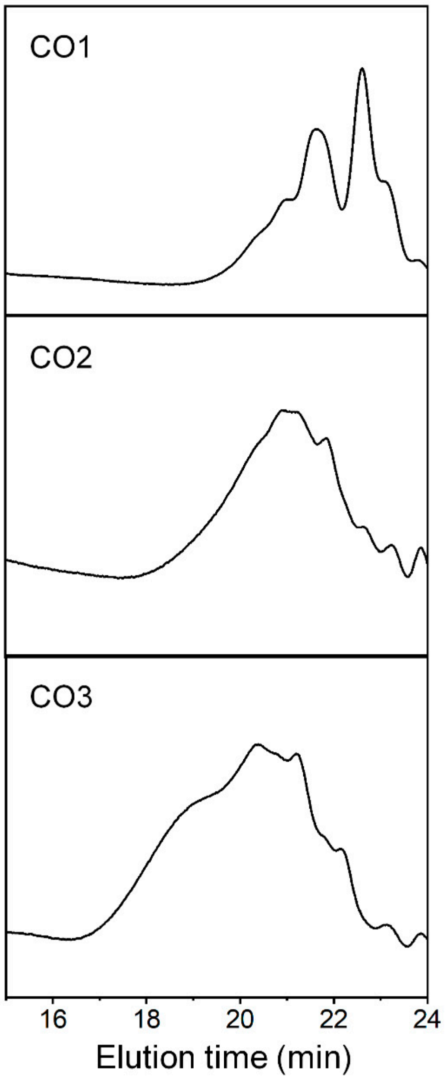

Figure S4. GPC curves of the CO1, CO2, and CO3.

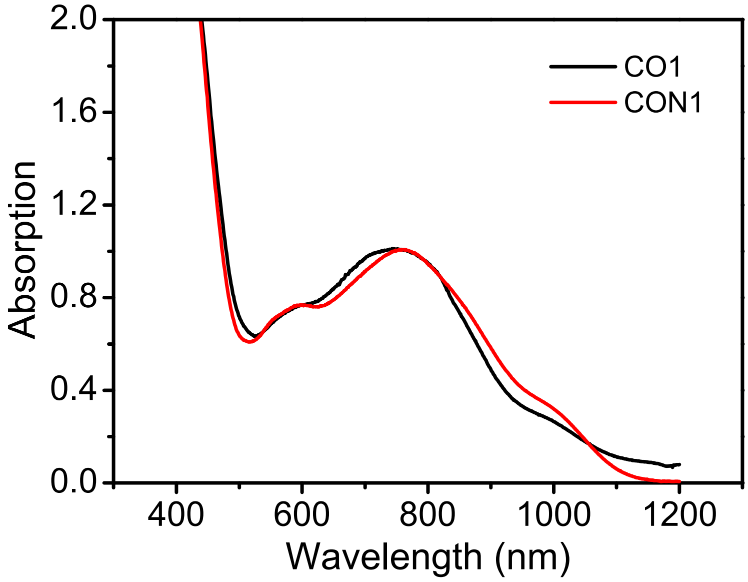

Figure S5. Normalized absorption spectra of CO1 and CON1.

Table S1. GPC results of the CO1, CO2, and CO3.

Sample name	Mn	Mw	PDI
CO1	3357	3734	1.11251
CO2	4063	5114	1.25857
CO3	5395	11043	2.04703
